# Thermal Properties of Calcium Sulphoaluminate Cement as an Alternative to Ordinary Portland Cement

**DOI:** 10.3390/ma14227011

**Published:** 2021-11-19

**Authors:** Małgorzata Gołaszewska, Barbara Klemczak, Jacek Gołaszewski

**Affiliations:** Faculty of Civil Engineering, Silesian University of Technology, Akademicka 5, 44-100 Gliwice, Poland; barbara.klemczak@polsl.pl (B.K.); jacek.golaszewski@polsl.pl (J.G.)

**Keywords:** hydration heat, isothermal heat flow, CSA cement, Portland cement, apparent activation energy

## Abstract

This paper presents the results of research into the heat of hydration and activation energy of calcium sulphoaluminate (CSA) cement in terms of the dependence on curing temperature and water/cement ratio. Cement pastes with water/cement ratios in the range of 0.3–0.6 were tested by isothermal calorimetry at 20 °C, 35 °C and 50 °C, with the evolved hydration heat and its rate monitored for 168 h from mixing water with cement. Reference pastes with ordinary Portland cement (OPC) were also tested in the same range. The apparent activation energy of CSA and OPC was determined based on the results of the measurements. CSA pastes exhibited complex thermal behaviour that differed significantly from the thermal behaviour of ordinary Portland cement. The results show that both the w/c ratio and elevated temperature have a meaningful effect on the heat emission and the hydration process of CSA cement pastes. The determined apparent activation energy of CSA revealed its substantial variability and dependence, both on the w/c ratio and the curing temperature.

## 1. Introduction

Environmental concerns are one of the most important challenges in current concrete technology, due to the high amount of CO_2_ emitted to the atmosphere during Portland clinker production. The production of 1 ton of clinker emits from 0.6 to 1 ton of CO_2_, depending on the applied technology [1,2]. To decrease the impact of the concrete industry on carbon dioxide emissions, many different approaches are currently taken, including decreasing the amount of Portland clinker in cement by its substitution with other materials such as blast furnace slags [3], pozzolans [4], fly ash [5], or limestone [6], or by using other binders not based on Portland clinker, such as geopolymers [7] or calcium sulphoaluminate (CSA) clinker [8,9].

CSA cement is a relatively new and uncommon type of cement, which was invented by Alexander Klein in the 1950s and introduced to industrial production in the 1960s [9]. CSA clinker is a hydraulic binder obtained by burning limestone, bauxite and gypsum [10]. Due to the lower amounts of limestone involved in the process, as well as lower burning temperature, it has lower CO_2_ emissions per 1 ton of clinker in comparison to Portland clinker [11]. Its phase composition, and thus its hydration process, differs significantly from Portland clinker. The mineral composition of CSA cement consists mostly of ye’elemite (C_4_A_3_Ŝ) with smaller amounts of belite (C_2_S) and calcium sulphate (CŜ), while Portland clinker consists mostly of alite (C_3_S), belite (C_2_S), tricalcium aluminate (C_3_A) and brownmillerite (C_4_AF). Therefore, instead of mostly CSH phases and portlandite (CH), as in Portland clinker, the main product of CSA clinker hydration is ettringite (C_6_AŜ_3_H_32_), stratlingite (C_2_AŜH_8_) and monosulphate, with low amounts of CSH also appearing due to the belite hydration process [12,13,14].

Due to ettringite being the main product of the hydration process, CSA cement is usually characterized by short initial and final setting times, high early strength, and low shrinkage [15,16]. Although all those properties are usually considered desirable in practical concrete technology, CSA cement is not widely used due to the high costs of the materials needed to produce the clinker, especially bauxite. However, with the increasing cost of Portland clinker production, this issue may prove to be less pressing. A more prevalent issue preventing the widespread use of CSA cement is the limited research on its properties, leading to an incomplete understanding of CSA cement as a material, especially in terms of the parameters used in the designing of concrete structures.

One of the relatively poorly recognized characteristics is its hydration heat and hydration heat development. Generally, the main reaction of CSA cement is connected to a rapid reaction of ye’elimite and gypsum, which form ettringite (AFt), monosulphate (AFm) and crystalline aluminium hydroxide (AH3), resulting in a rapid heat release and the high hydration heat main peak, usually in the first few hours after mixing cement with water [17,18,19]. Small amounts of belite present in the clinker react at a slower rate, forming limited amounts of the C-S-H phase, CH and gehlenite hydrate (C_2_ASH_8_) [20]. The research made by Winnefeld et al. [19] and Hargis et al. [17] has shown that the exact hydration heat development is dependent on the amount and form of the calcium sulphate added to the CSA clinker, with the addition of gypsum increasing hydration rate in comparison to anhydrite. It is therefore hard to draw comparisons between the hydration heat development between CSA cement and Portland cement.

In research by Li et al. [21], hydration heat evolution was characterized by both a higher hydration peak than in the case of Portland cement and its faster appearance. A similar effect was observed in research by Batog and Synowiec [22], Qin et al. [23] and Ramanathan [24]. However, the research of Li et al. [25] has shown that, while the main peak was higher, it could be later than in the case of Portland cement. The water–cement ratio also appears to affect the hydration heat of CSA. Research by Doval et al. [26] has shown that, with the increase in water-solids ratio (which was adopted in this research), the hydration heat can decrease. The same research found that, with higher temperatures, the hydration heat development is accelerated, and the hydration peak is greater. It must be noted, however, that those tests were performed for a very limited number of water-solids ratios (0.5, 0.7 and 1.0) that do not correspond well with water–cement ratios, which are more commonly used. The issue of choosing the right w/c ratio can prove to be more difficult in the case of CSA cement than Portland cement because, according to stoichiometric calculations, the right amount of water needed for the CSA cement to fully hydrate is 0.64, while for Portland cement it is less than 0.3 [14].

CSA hydration in a high curing temperature has been the subject of the research of Jeong et al. [27], in which hydration heat of CSA cement was tested at 30 °C, 60 °C and 90 °C. It has been found that the composition of the CSA cement plays an important role in the effect of temperature on the hydration, with the amount of ye’elemite in the cement affecting the response to the high temperature. In the case of higher ye’elemite content, the hydration heat at 90 °C was significantly lower than at 60 °C, while for the lower amount, with the increase of temperature the hydration heat also increased. Wang et al. [28] found that the hydration degree of CSA cement and clinker increases with increasing temperature. Additionally, with the increasing temperature, the type of hydration products changed in CSA clinker; however, in CSA cement no such effect was observed.

The thermal properties of cement, related to its hydration heat, are important in a variety of technological situations, such as concreting in low or high temperatures, and knowledge of these properties allows a better choice of cement for the given environmental situation. Hydration heat tests are the basis for the classification of cement as low heat (LH) or very low heat (VLH) cement according to the standard BS EN 197-1 [29]. Moreover, the time and size of the hydration peaks in the hydration flow charts can be the basics of understanding when to expect any heat phenomena, which can lead to better planning of the curing procedures.

Hydration heat development is also vital information needed to design concrete for a structure, also in the context of massive structures, where the high-temperature gradient between the centre of the structure and its surface may easily occur, leading to increased tensile stress in the element [30]. If the tensile stress induced by the uneven volume changes in the element, resulting from the temperature gradient exceeding the tensile strength of concrete, cracking may occur, with the simultaneous decrease in the overall load-bearing capacity of the element and loss of durability [31].

Moreover, high temperatures of the concrete can affect strength development at early stages [32]. Therefore, if the need arises to model the thermal behaviour of concrete and to assess its actual maturity, the equivalent age approach based on the Arrhenius concept is usually used. In this case, the value of apparent activation energy is required. Its value can be determined through both mechanical and calorimetric testing and subsequent calculations; however, the calorimetric method is often preferred [32,33,34,35,36]. In the case of CSA cement, this subject is poorly recognized.

The presented research focused on the hydration heat evolution of CSA cement pastes with different water/cement ratios by means of isothermal calorimetry at three different curing temperatures: 20 °C, 35 °C and 50 °C. Reference pastes with ordinary Portland cement (OPC) were also tested in the same range, which highlighted the distinct thermal behaviour of CSA cement. Next, the apparent activation energy of CSA and ordinary Portland cement was determined based on the results of the measurements. The effects of temperature and w/c ratio on the apparent activation energy based on the Arrhenius concept were also examined.

The addressed paper consists of five sections. After the introduction (Section 1), the experimental program with the basic characteristics of materials and testing methods are provided in Section 2. Next, the results obtained from the conducted study are presented and discussed in Section 3. Finally, the main conclusions are specified in Section 4.

## 2. Experimental Program

In the conducted research, commercially available CSA cement was used. A reference paste with ordinary Portland cement CEM I 42.5R (CEM I) was also tested. The chemical composition of the tested types of cement is presented in Table 1. The basic properties of both types of cement are depicted in Table 2.

Experimental tests were performed to study the effect of two factors influencing the hydration process: the water-cement ratio and the temperature. The applied water/cement ratio was equal to 0.3, 0.35, 0.4, 0.45, 0.5, 0.55 and 0.6, both for CSA and for CEM I cement. The chosen w/c ratios reflect the commonly used proportion between cement and water.

Three curing temperatures were used to measure the evolved heat. A temperature of 20 °C was applied as a reference value, typically used in heat of hydration studies. Additionally, the influence of increased curing temperatures (35 °C, 50 °C) on the hydration process of CSA cement was investigated. The curing temperature of 35 °C can easily occur in concrete structures cast in the summer season, while 50 °C may be applied to structures of larger cross-sectional dimensions with internal self-heating and elevated curing temperature, directly affecting the amount of heat released. Furthermore, the heat development at these three curing temperatures enabled the determination of the activation energy, which is described later in this section.

Hydration heat was measured in an isothermal calorimeter, TAM Air. The tests were performed according to the European standard PN-EN 196-11 [37] with the internal mixing procedure, allowing for the measurement of heat development from the moment of adding water to cement. As a reference, quartz sand was used, in an amount calculated based on its heat capacity corresponding to the cement paste samples.

The measurement was conducted on cement paste samples consisting of 5 g of CSA or CEM I cement and distilled water in an amount calculated from the assumed w/c ratio. The ampoules with cement and water were inserted into the calorimeter at least 24 h before the start of the measurement so that the temperature of the sample was equal to the set temperature (20 °C, 35 °C or 50 °C) at the start of the procedure. The measurement lasted 168 h from the time of adding water to the cement. Measurements for each instance were performed three times, and the mean value was calculated. For all of the instances, the standard deviation was lower than 6%.

Subsequently, based on the obtained results of the released heat at different temperatures, the activation energy of the tested types of cement was determined. The apparent activation energy is a crucial measure in the equivalent age approach used to estimate the combined effects of time and temperature on the property development of cementitious materials. This approach is a simple and common method for a reliable prediction of in-place strength during the construction of structures. Furthermore, the hydration heat development at different curing temperatures can also be accounted for, which is important in massive structures.

Hence, the sensitivity of the cement hydration process to the curing temperature can be determined using a function introduced by Hansen and Pedersen [32] which is based on the Arrhenius rate concept, originally describing the effect of temperature on the rate of a chemical reaction [34,38]. Following this function, the equivalent age, $t_{eq}$, is computed as follows:

(1) $$t_{eq}=\int_{0}^{t}e^{\frac{-E_{a}}{R}\left(\frac{1}{T\left(\tau \right)}-\frac{1}{T_{ref}}\right)}d\tau$$

with:

*T*(*τ*), the actual temperature of the cementitious material, °C;
*T_ref_*, the reference temperature usually equal to 20 °C;
*R*, the universal gas constant equals 8.314 J mol^−1^·°C^−1^;
$E_{a}$, the activation energy, J mol^−1^;
τ, time, h or day.

The application of Equation (1) involves the conversion of the actual age of the cementitious material to its equivalent age at the reference temperature. The temperature dependence in Equation (1) is described by the value of the apparent activation energy, $E_{a}$, thus, its value is essential. The reported experimental tests revealed its dependence on the cement type, curing temperature and also, to a lesser extent, on the water/cement ratio [34,39,40,41]. Although the activation energy can be determined by strength tests of samples curing at different temperatures [32,42], isothermal calorimetry is appraised as a better method to quantify the activation energy [33,35]. Considering the computation of the activation energy based on the isothermal tests, the different procedures are discussed in [35,42]. However, regardless of the method of its determination, it should be remembered that only an approximate description of the influence of temperature on cement hydration is obtained, which is reflected in the name ‘‘apparent’’ of the determined activation energy [33].

In this study, the rate method [43,44,45] has been applied to determine the discussed energy. Following this method, the apparent activation energy, $E_{a}$, is calculated based on heat evolution rates *q*_1_ and *q*_2_, consistent with the same evolved heat, *Q_i_*, and two curing temperatures, $T_{1}$ and $T_{2}$:

(2) $$E_{a}\left(Q_{i}\right)=-\frac{R}{\frac{1}{T_{1}\left(Q_{i}\right)}-\frac{1}{T_{2}\left(Q_{i}\right)}}ln\left[\frac{q_{1}\left(Q_{i}\right)}{q_{2}\left(Q_{i}\right)}\right]$$

## 3. Results and Discussion

### 3.1. Evolution and the Rate of Hydration Heat

**Figure 1:**
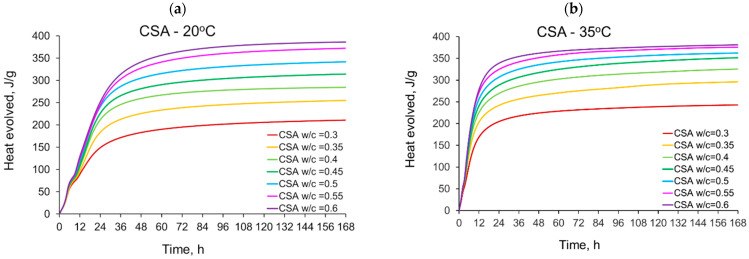

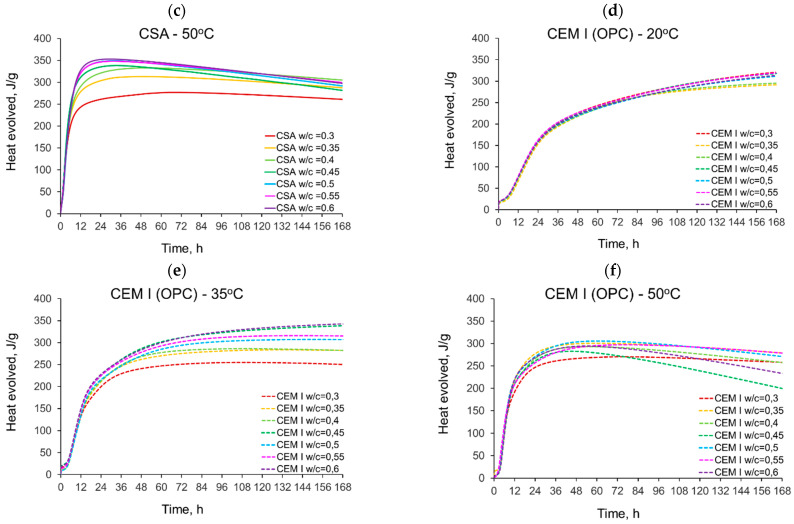
The heat evolved from cement pastes with different water/cement ratios in isothermal tests at different curing temperatures: (**a**) CSA at 20 °C, (**b**) CSA at 35 °C, (**c**) CSA at 50 °C, (**d**) CEM I (OPC) at 20 °C, (**e**) CEM I (OPC) at 35 °C, (**f**) CEM I (OPC) at 50 °C.

**Figure 2:**
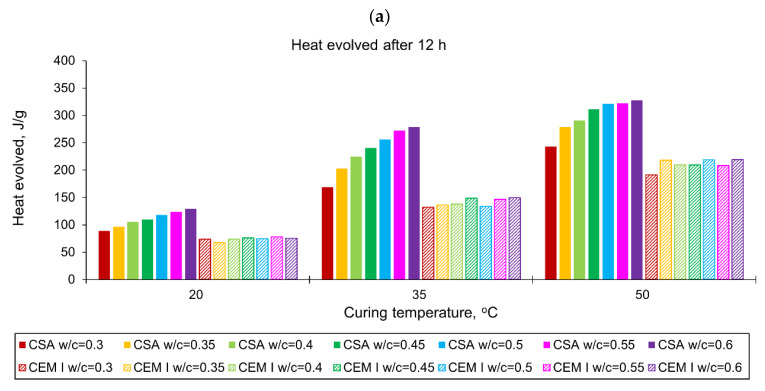

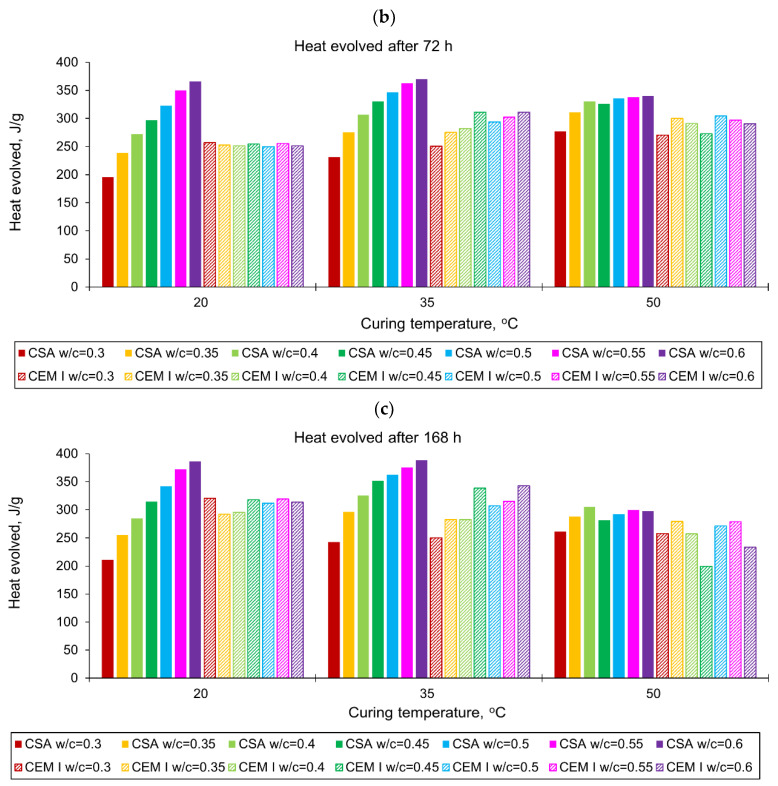
The heat evolved from CSA and CEM I (OPC) cement pastes with different w/c ratios in isothermal tests at different curing temperatures after: (**a**) 12 h, (**b**) 72 h, (**c**) 168 h.

The curves of the heat evolution for CSA and CEM I (OPC) cements with different water/cement ratios measured in isothermal tests at 20 °C, 35 °C and 50 °C are shown in Figure 1. The values of the released heat are listed in Table 3 (CSA) and Table 4 (OPC). To facilitate the interpretation of the obtained values of the released heat, they are also presented graphically in Figure 2.

Generally, a significant impact of the water/cement ratio on the amount of the released heat of CSA cement can be noticed. For all applied curing temperatures, the evolved heat increases with the increase of the w/c ratio (Figure 1a–c). In the initial period of 12 h, this effect is not so meaningful, and the heat emission increases about 6% for each w/c ratio increase of 0.05. In the later period, after 41, 72 and 168 h, the average value of heat growth is 10% under the curing temperature equal to 20 °C and 35 °C. Simultaneously, the greatest increase in heat release can be seen between CSA pastes with a water/cement ratio of 0.3 and 0.35 (Figure 1a,b). This is over 20%, considering the heat released after 41, 72 and 168 h. For higher values of w/c, this growth successively decreases. For w/c = 0.6, it is only 4% and 1.5% for tests at 20 °C and 35 °C, respectively.

Considering ordinary Portland cement, CEM I, the water/cement effect is not so significant and clear as in CSA pastes (Figure 2). In detail, at a curing temperature of 20 °C, the heat released is practically the same for different w/c. Slight differences are visible only after 120 h, and they are less than 3% (Figure 1d). At a higher curing temperature of 35 °C, the w/c effect is visible, but it is still less pronounced than for CSA cement (Figure 1e). In this case, after 168 h, the greatest growth (12%) in the released heat was observed between OPC pastes with a w/c of 0.3 and 0.35 (Figure 1e).

The same observations can also be made for isothermal tests at 50 °C (Figure 1c,f), but in this case, the heat-releasing characteristic revealed unexpected thermal behaviour of the tested pastes. In this context, the typical progress of heat evolution is rapid growth during the initial period of hydration and final stabilization as hydration slows. Such development of the hydration heat is visible for curing temperatures of 20 °C and 35 °C (Figure 1a,b,d,e), while at 50 °C, a decrease in the amount of heat released is noticed (Figure 1c,f). The time after which the amount of released heat begins to decrease depends on the water/cement ratio of CSA paste and reaches 68 h for a w/c of 0.3, and only 30 h for a w/c of 0.6. For OP cement (CEM I) this time is delayed to 72 h, except for the paste with a w/c equal to 0.45. Various reasons for such development of the heat may be considered.

The first reason may be an incorrect measurement or a defect in the measuring device. This was eliminated by repeated measurements which showed the same results. Next, the decreased amount of the released heat may be connected to the stopped hydration process; however, isothermal calorimetry cannot precisely explain this phenomenon. Furthermore, the most probable reason for the decrease in the released heat is due to the technical issue of water evaporation in the samples, which can occur at higher temperatures. The process of evaporation requires energy, which causes the temperature of the sample to drop, and therefore to show lower heat values. The process may have been occurring from the start of the test, but with the high amount of heat produced in the exothermic reaction of cement with water, it was not possible to notice the lowering of the generated heat. This claim can be substantiated by the fact that the higher the w/c ratio of the tested CSA cement pastes, the higher the difference in the heat after 168 h between samples cured at 20 °C and 50 °C. In the case of low w/c ratios, between 0.3 and 0.4, the heat loss is minimal, and the heat produced during the first 168 h of hydration by samples cured at 50 °C is slightly higher than that of samples cured at 20 °C. With the increasing w/c ratio, the difference between the cumulative heat after 168 h of samples cured at 50 °C and 20 °C increases, with samples cured at 50 °C reaching much lower values, up to over 30% lower in the case of a w/c ratio of 0.6. This effect can be connected to a significantly higher amount of water available for the process of evaporation. It should be noted that the CSA cement requires a high amount of water for the reaction of hydration to fully take place. According to stoichiometric calculations, CSA clinker requires a high w/c ratio of over 0.6 to fully react [13], and therefore, for lower w/c ratios, more water may be chemically bound into ettringite.

Considering the overall effect of temperature on the amount of heat released in CSA pastes, a substantial increase in heat emission for the hydration temperature rise from 20 °C to 35 °C or 50 °C can be observed. Moreover, hydration in the first hours is more sensitive to temperature. After 12 h, the evolved heat is nearly two times higher at 35 °C and 50 °C, comparing the corresponding values of heat measured at 20 °C. Later, this is about 6–20%, with the lowest values for 168 h. It can be remarked that the increase in the amount of heat released under the higher curing temperature slows for the higher w/c. The temperature also influences the hydration of OP cement, but this effect is less than in the case of CSA cement.

Finally, comparing the amount of the heat released after 168 h and at 20 °C, OPC pastes are characterized by higher values for w/c equal to 0.3–0.45 (Figure 2c). In the range of w/c = 0.5–0.6, the heat evolved by CSA pastes is higher, especially in the case of w/c = 0.6; 73 J/g was more evolved in the CSA paste. At the same time, at the curing temperature equal to 35 °C, only in the case of w/c equal to 0.3 was more heat released in OP cement; in other w/c, CSA cement was characterized by higher evolved heat (from 7 J/g to 60 J/g).

**Figure 3:**
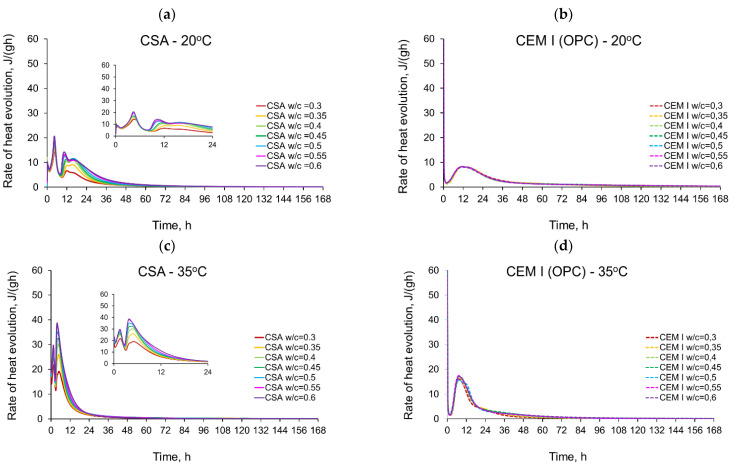

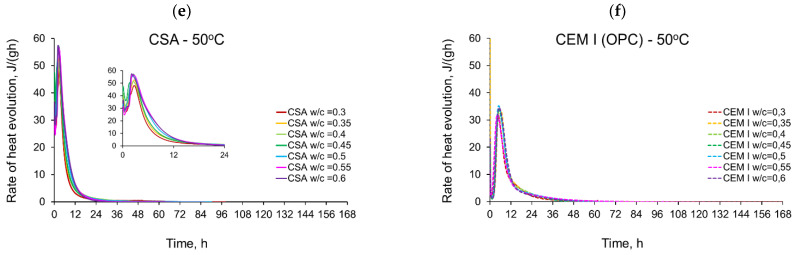
Rate of heat evolution of cement pastes with different water/cement ratios in isothermal tests at different curing temperatures: (**a**) CSA at 20 °C, (**b**) CEM I (OPC) at 20 °C, (**c**) CSA at 35 °C, (**d**) CEM I (OPC) at 35 °C, (**e**) CSA at 50 °C, (**f**) CEM I (OPC) at 50 °C.

The rate of the hydration heat measured for all tested CSA and OPC pastes in isothermal tests at 20 °C, 35 °C and 50 °C are depicted in Figure 3 and summarized in Table 3 and Table 4. All curves plotted in Figure 3 show the distinct behaviour of CSA pastes compared to CEM I (OPC) paste. The main differences are in the number of exothermic peaks, their values and the occurring time. The several heat flow peaks in CSA cement are reported in other tests [19,46,47], and the results from the presented study confirm this tendency.

It should be noted that there are significant differences in the behaviour of OPC and CSA cements, which can be traced to their different hydration processes. OPC cement hydration is based on the reaction of four main clinker phases: alite C_3_S, belite C_2_S, tricalcium aluminate C_3_A and brownmillerite C_4_AF, with water, producing C-S-H gel and portlandite [48]. The reaction is usually characterized by a high heat evolution in the first several minutes from adding water to the cement, due to dissolution and C_3_A reaction with gypsum, resulting in ettringite. After that, the reaction slows down for up to several hours (induction phase), to restart with alite hydration, followed by the belite and C_3_A hydration [46,48]. In the case of CSA cement, the main constituents of the CSA clinker are ye’elimite and gypsum, with low amounts of belite present. The process of hydration of CSA cement is swift and results mostly in the production of ettringite (AFt), monosulphate (AFm), crystalline aluminum hydroxide (AH_3_) and stratlingite (C_2_ASH_8_), with small amounts C-S-H phase appearing late in hydration process due to belite reaction [46]. This results in a significantly faster start of the reaction than in the case of OPC, a short induction phase and high hydration peaks [18,20,25].

Considering the isothermal tests of CSA at 20 °C (Figure 3a), four peaks with values depending on the w/c ratio can be seen. The first peak in the initial period occurs at 0.25 h and reaches 8–10 Jg^−1^·h^−1^. Contrary to the CEM I cement paste (Figure 3b), this initial maximum represents the smallest value in all peaks detected in the curve of the heat release rate. The first peak may be attributed to the dissolution of the solid grains in water [48]. It has been noted that the CEM I dissolution peak might be higher due to the extremely fast C_3_A dissolution and the production of ettringite in a reaction that differs greatly from the one taking place in the CSA cement [46]. Next, a certain induction period can be observed in the case of both the CSA cement and CEM I. It should be noted that, while the reaction of CSA cement is slow during this period, the hydration of ye’elemite is progressing, as the heat flow rate is much higher than in the case of CEM I, and in 50 °C the induction period cannot be clearly separated from the initial peak connected with dissolution. The induction phase of Portland cement is longer than that of CSA cement at all temperatures; at 20 °C it is twice as long, and at 35 °C it is around 5 times longer, which points to a faster reaction speed of the phases present in CSA cement.

The second peak in CSA cement takes the highest values (14.2–20.7 Jg^−1^·h^−1^), increasing with the increase of the water/cement ratio. The time of its occurrence is almost identical and equal to 4.5 h for all CSA pastes. The peak is connected to the initial dissolution of the sulphates and the consequent reaction of calcium sulphoaluminate, anhydrite and the water in which ettringite is formed [48].

The rate of hydration heat of CSA at the third local maximum is lower (6.6–14.3 Jg^−1^·h^−1^) and occurs at a similar time as the maximum for CEM I paste. The third local maximum appears after 10.5–12 h, which is earlier the higher the w/c. After the third peak, the rate of reaction gently falls, and the fourth local maximum with a strength similar to the third peak (5.9–11.7 Jg^−1^·h^−1^) appears after the next 3–4 h. The third peak is a result of the main reaction of main ettringite production, followed by depletion of the gypsum and the formation of monosulphates [18]. In the case of 20 °C, a fourth peak can be observed, which may be attributed to the second ettringite formation or point to the possibility that the monosulphate formation in 20 °C starts later than during the main peak of ettringite reaction; however, in higher temperatures, this process is accelerated and does not show separately in the heat flow [19,20].

When the curing temperature increases from 20 °C to 35 °C, the induction period is significantly shortened for all tested CSA pastes (Figure 3c). Consequently, the time of the second exothermic peak is shortened to 1.4–1.7 h, and for the third peak to 3.7–4.0 h. The second and third exothermic peaks show a very narrow shape, with much higher values compared to 20 °C. Contrary to the isothermal tests at 20 °C, the third peak revealed greater values (17.0–38.7 Jg^−1^·h^−1^) than the second peak (21.8–29.7 Jg^−1^·h^−1^), except for pastes with w/c ratio equal to 0.3 and 0.35. A clear fourth maximum was not observed for tests at 35 °C.

Similar observations for CSA cement may be seen for the isothermal tests at 50 °C (Figure 3e). The strength of the second and third peaks seriously increases, and for all water/cement ratios the third peak takes the highest value. The third exothermic effect grows from 4.0 (w/c = 0.6) to 7.3 (w/c = 0.3) times, with the curing temperature increasing from 20 °C to 50 °C. Thus, the rate of reaction increases rapidly, reaching maximum values at a time of 0.08–0.17 h (second peak) and 2.1–2.7 h (third peak) after initial mixing. Simultaneously, in the case of CEM I, the maximum value of the exothermic peak occurs at 6 h and increases by about 4 times at 50 °C (Figure 3f).

It should be noted that the presence of additional peaks of significant power in the case of CSA cement is complex and of significant importance. On the one hand, it accelerates the maturation process and the development of the mechanical properties of the cementitious material, which is generally a desirable feature. On the other hand, the maturation of the cement material at a too high initial curing temperature may damage its structure and, consequently, reduce its final strength [49], despite its initially rapid growth.

Furthermore, the large amount of rapidly released heat and the prolonged heat release time to the third maximum may cause numerous problems such as long-term elevated hardening temperatures in the structure, which often initiates early thermal cracks.

### 3.2. Apparent Activation Energy

**Figure 4:**
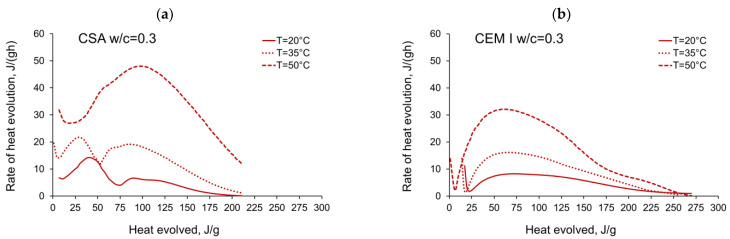
Rate of heat evolution in the function of heat evolved for cement pastes with w/c = 0.3: (**a**) CSA, (**b**) CEM I (OPC).

**Figure 5:**
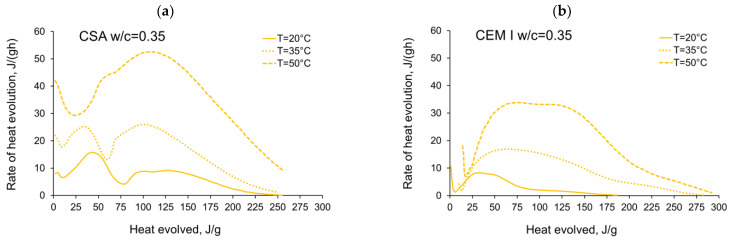
Rate of heat evolution in the function of heat evolved for cement pastes with w/c = 0.35: (**a**) CSA, (**b**) CEM I (OPC).

**Figure 6:**
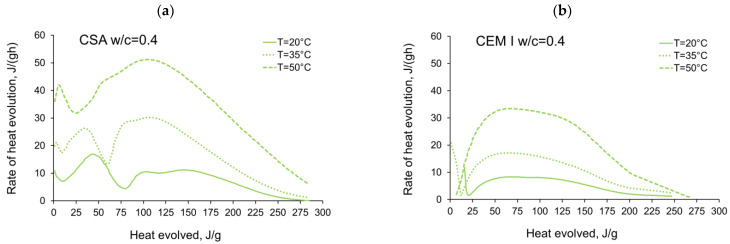
Rate of heat evolution in the function of heat evolved for cement pastes with w/c = 0.4: (**a**) CSA, (**b**) CEM I (OPC).

**Figure 7:**
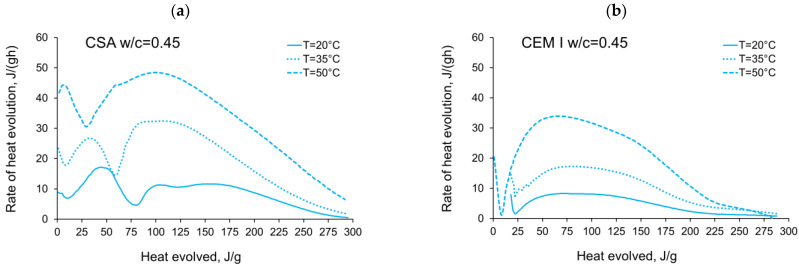
Rate of heat evolution in the function of heat evolved for cement pastes with w/c = 0.45: (**a**) CSA, (**b**) CEM I (OPC).

**Figure 8:**
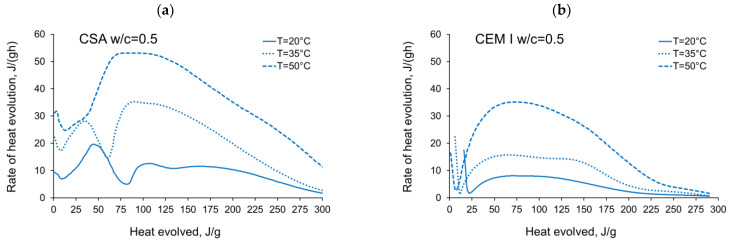
Rate of heat evolution in the function of heat evolved for cement pastes with w/c = 0.5: (**a**) CSA, (**b**) CEM I (OPC).

**Figure 9:**
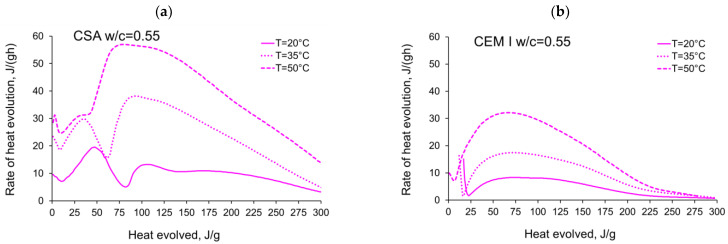
Rate of heat evolution in the function of heat evolved for cement pastes with w/c = 0.55: (**a**) CSA, (**b**) CEM I (OPC).

**Figure 10:**
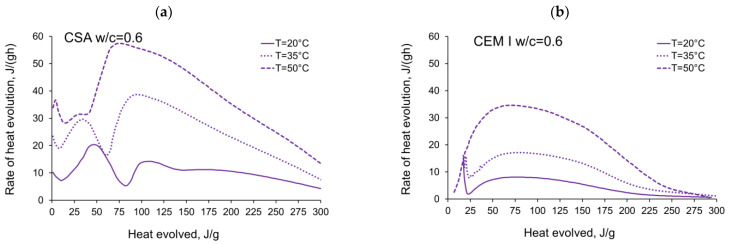
Rate of heat evolution in the function of heat evolved for cement pastes with w/c = 0.6: (**a**) CSA, (**b**) CEM I (OPC).

The activation energy was calculated for all tested CSA cement pastes with different water/cement ratios as well as for reference pastes with ordinary Portland cement, CEM I. Two sets of the results from isothermal calorimetry are used in Equation (2): 20 °C/35 °C and 20 °C/50 °C. The necessary development of the heat evolution rates needed for Equation (2), *q*(20 °C), *q*(35 °C) and *q*(50 °C) related to the same amount of the evolved hydration heat, is depicted in Figure 4, Figure 5, Figure 6, Figure 7, Figure 8, Figure 9 and Figure 10, respectively for all tested w/c. These diagrams highlight once again the different thermal behaviours of CSA compared to OPC (CEM I).

**Figure 11:**
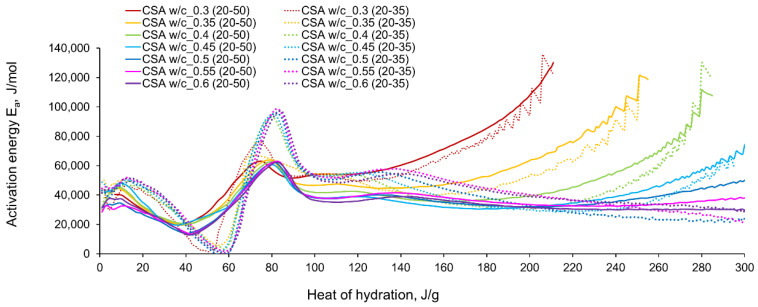
The apparent activation energy of CSA cement pastes with different w/c ratios.

**Figure 12:**
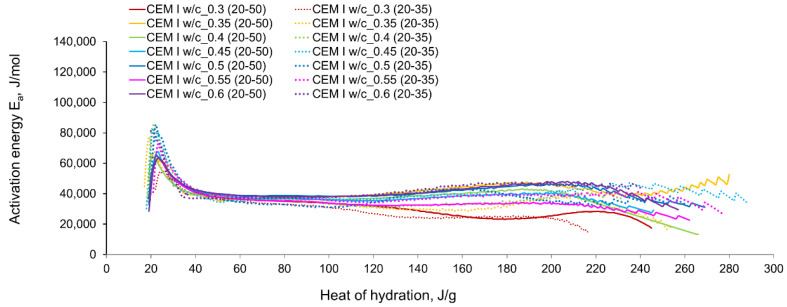
The apparent activation energy of CEM I (OPC) cement pastes with different w/c ratios.

The computed evolution of activation energy for CSA and CEM I cement pastes with different water/cement ratios can be seen in Figure 11 and Figure 12, respectively. The following observations can be perceived from the apparent activation energy evolution:

- The variations in apparent activation energy of CSA cement pastes are quite substantial, especially compared to the relatively constant values obtained for reference cement pastes made of ordinary Portland cement, CEM I. Moreover, in the case of CSA cement, both water/cement ratio and curing temperature affect the values and the evolution of apparent activation energy.
- For CSA cement, the $E_{a}$ variations are qualitatively similar considering the two applied sets of curing temperatures (20 °C/35 °C and 20 °C/50 °C); however, they vary quantitatively. In the case of ordinary Portland cement, CEM I, the differences between the two sets of curing temperatures are not so substantial,
- In the case of CSA, for released heat lower than 100 J/g, particularly rapid changes in the values of activation energy are visible, and no stable period is observed. Moreover, these variations are more distinctive for the activation energy calculated using the isothermal tests at 20 °C/35 °C. In this case, the maximum value of $E_{a}$ reached nearly 100,000 Jmol^−1^ for the hydration heat equal to ~80 J/g, while earlier the activation energy almost dropped to 400–1000 Jmol^−1^ (for heat evolved close to 60 J/g). This effect can already be seen in the graphs presenting the rate of heat released at 20 °C and 35 °C (Figure 4, Figure 5, Figure 6, Figure 7, Figure 8, Figure 9 and Figure 10), where the overlap of curves is noticeable in the range of hydration heat 50–60 J/g. For hydration heat greater than 100 J/g, the curves describing the activation energy obtained from two sets of curing temperatures (20 °C/35 °C and 20 °C/50 °C) are more congruous. Simultaneously, the influence of the water/cement ratio is perceivable. In this regard, for w/c ≤ 0.45, the activation energy successively grows up to the value of 120,000 J/mol^−1^. For w/c equal to 0.5 and 0.55, the increase of the energy value is insignificant, and for w/c = 0.6, a decrease is even visible.

**Figure 13:**
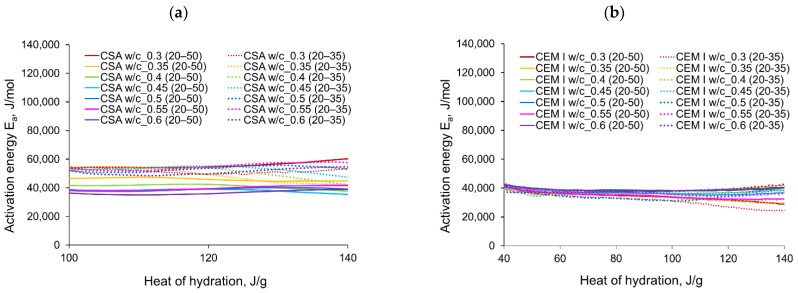
The apparent activation energy in its stable range: (**a**) CSA, (**b**) CEM I (OPC).

For CSA cements, with the fairly wide variations observed in the range of hydration heat 0–100 J/g and the previously described subsequent growth, the evolution curves of $E_{a}$ present a very limited stable period. The mean values of the activation energy obtained for its stable region for CSA cement with different w/c are shown in Table 5. Generally, the greater mean value of activation energy in the considered stable range 101–140 J/g is obtained from the isothermal tests performed at 20 °C/35 °C, except for the cement paste with w/c = 0.3. The values of apparent activation energy for the considered range are also plotted in Figure 13a.

For ordinary Portland cement, a range with an almost stable value of $E_{a}$ is between 101–140 J/g (Figure 13b). Contrary to CSA, the slightly greater values of the activation energy are visible from the tests at 20 °C/50 °C. The average values in the stable period for CSA cement are much higher than for ordinary Portland cement. The resulting values are:

- for CSA: 51,900 J/mol (tests 20 °C/35 °C) and 42,213 J/mol (tests 20 °C/50 °C),
- for OPC: 35,424 J/mol (tests 20 °C/35 °C) and 37,228 J/mol (tests 20 °C/50 °C).

## 4. Conclusions

In this study, the hydration heat of CSA cement pastes with different water/cement ratios has been studied by isothermal tests at different curing temperatures and compared to the ordinary Portland cement. Based on the Arrhenius concept, the coupled effects of temperature and w/c on the apparent activation energy are also examined. The main results can be listed as follows:

- The evolved heat of CSA cement pastes increases with the increase of the water/cement ratio. The largest, 20% increase in heat emission, was recorded for the water/cement change from 0.3 to 0.35. For higher w/c ratios the average difference between consecutive measured w/c ratios was 10%. In the case of OPC, the heat released at the curing temperature of 20 °C is practically the same for different w/c. At a higher curing temperature of 35 °C, the w/c effect is visible but still less pronounced than for CSA cement.
- The temperature of curing affects the evolved heat of CSA cement the most in the first 12 h. After 168 h, there is no significant difference between the evolved heat at 20 °C and 35 °C, while the evolved heat at 50 °C was significantly lower, which is most likely due to water vaporization. Nevertheless, the hydration of CSA pastes is significantly accelerated at 35 °C and 50 °C. The effect of the elevated temperature on the hydration process is much greater than that of ordinary Portland cement.
- The complex thermal behaviour of CSA pastes with several heat flow maxima has been confirmed in the performed isothermal tests at different curing temperatures. Four heat flow peaks in CSA cement are detected for tests at 20 °C, while at 35 °C and 50 °C three peaks are confirmed. Generally, the strength of the hydration peaks decreased with decreasing water/cement ratio. At 35 °C and 50 °C, the end of the induction period and the occurring time of the main peaks are remarkably shortened. Simultaneously, the exothermic peaks have extremely narrow shapes, and the peak value increases greatly compared to tests at 20 °C.
- The variations in apparent activation energy are substantial, and they cannot be considered as a constant value for the CSA type of cement. Only for the relatively small range of the evolved heat (101–140 J/g) did the curves of energy evolution reveal steady behaviour. In this case, the average activation energy depends both on the water/cement ratio and the curing temperature. The average values in the stable period for CSA cement are much higher than those for ordinary Portland cement.
- The hydration region of CSA with especially rapid variations of activation energy covers the initial period of heat release, up to the value of 100 J/g. Using the isothermal results at 20 °C and 35 °C in the calculation procedure, especially sharp changes in energy are obtained. Moreover, both very small (400–1000 J·mol^−1^) and extremely high (100,000 Jmol^−1^) values are observed.

To summarize, the conducted tests indicated a different thermal behaviour of CSA cement compared to ordinary Portland cement. Calcium sulphoaluminate cement is characterized by the rapid course of the hydration process and significant amounts of heat being released in the initial period of hydration. These properties should be given special attention to when choosing the practical application of the CSA cement. The high variability of the activation energy and the impossibility of determining its relatively constant value can lead to problems when using the equivalent age approach for predicting the CSA composites’ strength development at elevated curing temperatures.

## Figures and Tables

**Table 1:** Composition of CSA and CEM I (OPC) cements.

Cement Type	Constituent, %									
	LOI	SiO_2_	Al_2_O_3_	Fe_2_O_3_	CaO	MgO	SO_3_	Na_2_O	K_2_O	Na_2_O_eq_
CSA	0.46	9.2	28.1	1.52	39.2	3.5	11.4	0.08	0.35	-
CEM I (OPC)	-	20.6	4.7	2.8	64.4	1.2	2.8	0.2	0.4	0.46

**Table 2:** Basic properties of CSA and CEM I (OPC) cements.

Cement Property	Unit	Value	
		CSA	CEM I (OPC)
Initial setting time	min	50	191
The soundness of cement, by Le Chatelier’s method	mm	1	0.4
Compressive strength after 2 days	MPa	42.6	30.1
Compressive strength after 28 days	MPa	67.7	57.4
Specific surface area	cm^2^/g	5600	4400

**Table 3:** Characteristic values of the heat evolved in CSA cement pastes.

Sample, Curing Temperature	The Heat Evolved, Jg^−1^				The Peak Value of Heat Evolution Rate, Jg^−1^·h^−1^		
	12 h	41 h	72 h	168 h	Second Peak	Third Peak	Fourth Peak
CSA w/c = 0.3							
20 °C	88.9	177.3	195.3	211.0	14.2	6.6	5.9
35 °C	168.7	220.8	231.7	243.0	21.8	19.2	-
50 °C	243.1	270.0	276.9	261.1	35.5	48.0	-
CSA w/c = 0.35							
20 °C	96.5	219.4	238.9	255.2	15.7	9.3	9.2
35 °C	203.0	260.3	275.5	296.2	25.1	26.1	-
50 °C	278.9	312.6	311.0	287.5	40.1	51.0	-
CSA w/c = 0.4							
20 °C	105.6	253.0	272.3	284.6	16.8	10.5	11.2
35 °C	225.1	290.6	307.2	325.4	26.5	30.2	-
50 °C	291.0	331.7	330.6	305.0	41.0	51.2	-
CSA w/c = 0.45							
20 °C	109.5	273.9	296.5	314.4	17.1	11.3	11.6
35 °C	240.4	311.6	330.5	351.6	26.8	32.4	-
50 °C	311.5	337.2	326.0	281.6	47.1	57.2	-
CSA w/c = 0.5							
20 °C	118.1	296.0	322.4	341.8	19.2	12.6	11.7
35 °C	255.8	330.0	346.8	362.5	28.1	35.3	-
50 °C	321.4	347.6	336.3	291.8	33.9	56.3	-
CSA w/c = 0.55							
20 °C	124.3	315.7	350.1	372.3	19.5	13.2	10.9
35 °C	271.8	346.6	362.7	375.8	29.4	38.5	-
50 °C	322.0	347.9	337.8	299.5	31.2	56.8	-
CSA w/c = 0.6							
20 °C	129.2	327.9	365.8	386.4	20.7	14.3	11.2
35 °C	278.5	358.1	369.9	388.3	29.7	38.7	-
50 °C	327.4	351.1	340.3	297.6	36.7	57.4	-

**Table 4:** Characteristic values of the heat evolved in CEM I (OPC) cement pastes.

Sample, Curing Temperature	The Heat Evolved, Jg^−1^				The Peak Value of Heat Evolution Rate, Jg^−1^·h^−1^		
	12 h	41 h	72 h	168 h	Second Peak	Third Peak	Fourth Peak
CEM I w/c = 0.3							
20 °C	73.8	213.6	257.0	320.8	8.2	-	-
35 °C	132.1	235.2	250.9	250.2	16.1	-	-
50 °C	191.3	263.8	270.4	258.0	32.1	-	-
CEM I w/c = 0.35							
20 °C	67.5	204.7	252.4	291.6	8.3	-	-
35 °C	136.5	254.2	275.3	282.4	16.9	-	-
50 °C	218.4	297.2	299.9	279.1	33.9	-	-
CEM I w/c = 0.4							
20 °C	73.9	206.1	251.3	295.4	8.3	-	-
35 °C	137.6	258.9	282.1	282.3	17.1	-	-
50 °C	209.6	290.4	291.2	257.7	33.5	-	-
CEM I w/c = 0.45							
20 °C	76.1	211.3	254.3	317.8	8.3	-	-
35 °C	148.5	273.3	311.4	338.6	17.2	-	-
50 °C	209.5	282.3	272.8	199.8	33.9	-	-
CEM I w/c = 0.5							
20 °C	74.8	208.2	249.7	311.5	8.1	-	-
35 °C	133.9	257.7	294.0	306.9	15.7	-	-
50 °C	218.7	299.5	304.7	271.1	35.2	-	-
CEM I w/c = 0.55							
20 °C	78.0	214.3	255.4	319.3	8.3	-	-
35 °C	146.6	266.6	302.1	315.1	17.5	-	-
50 °C	208.4	285.4	297.0	278.7	32.1	-	-
CEM I w/c = 0.6							
20 °C	75.7	209.7	251.2	313.5	8.1	-	-
35 °C	149.7	271.2	311.2	342.6	17.1	-	-
50 °C	219.2	290.9	290.4	233.4	34.6	-	-

**Table 5:** The value of *E_a_* calculated for its nearly stable value.

Value	Apparent Activation Energy, J mol^−1^						
	w/c = 0.3	w/c = 0.35	w/c = 0.4	w/c = 0.45	w/c = 0.5	w/c = 0.55	w/c = 0.6
Average values calculated from tests 20 °C/35 °C							
CSA—period	in range of evolved heat: 101–136 Jg^−1^						
CSA—value	50,917	49,131	51,623	53,477	53,230	53,906	51,016
CEM I (OPC)—period	in range of evolved heat: 40–140 Jg^−1^						
CEM I (OPC)—value	31,211	33,737	34,638	36,667	36,615	36,820	38,282
Average values calculated from tests 20 °C/50 °C							
CSA—period	in range of evolved heat: 101–136 Jg^−1^						
CSA—value	54,978	45,968	41,158	38,169	38,915	39,712	36,592
CEM I (OPC)—period	in range of evolved heat: 40–140 Jg^−1^						
CEM I (OPC)—value	33,922	38,208	37,085	36,805	39,010	36,601	38,967

## Data Availability

Data is contained within the article.

## References

[1] Favier A., De Wolf C., Schrivener K., Habert G. (2018). A Sustainable Future for the European Cement and Concrete Industry: Technology Assessment for Full Decarbonisation of the Industry by 2050.

[2] Skjærseth J.B., Eikeland P.O. (2016). Corporate Responses to EU Emissions Trading: Resistance, Innovation or Responsibility.

[3] Jain M. (2014). Use and properties of blast furnace slag as a building material—A review. Int. J. Recent Contrib. Eng. Sci. IT.

[4] Lemougna P.N., Wang K.-T., Tang Q., Nzeukou A., Billong N., Melo U.C., Cui X.-M. (2018). Review on the use of volcanic ashes for engineering applications. Resour. Conserv. Recycl..

[5] Giergiczny Z. (2019). Fly ash and slag. Cem. Concr. Res..

[6] Wang D.H., Shi C.J., Farzadnia N., Shi Z.G., Jia H.F., Ou Z.H. (2018). A review on use of limestone powder in cement-based materials: Mechanism, hydration and microstructures. Constr. Build. Mater..

[7] Juenger M.C.G., Winnefeld F., Provis J.L., Ideker J.H. (2011). Advances in alternative cementitious binders. Cem. Concr. Res..

[8] Winnefeld F. Calcium sulfoaluminate cement: An example of a low CO_2_—Alternative to portland cement. Proceedings of the WTA-Colloquium—Effect of Climate Change on Built Heritage.

[9] Kubissa W. (2020). Air permeability of air-entrained hybrid concrete containing CSA cement. Buildings.

[10] Nguyen K.S., Nguyen-Ngoc T.H., Nguyen-Phung A.T., Do Q.M. Preparation of calcium sulfoaluminate cement from baux-ite/red mud of Tan Rai—Lam Dong. Proceedings of the 7th International Conference of Asian Concrete Federation ACF 2016.

[11] Miller S.A., Horvath A., Monteiro P.J.M. (2016). Readily implementable techniques can cut annual CO_2_ emissions from the production of concrete by over 20%. Environ. Res. Lett..

[12] Chen I.A., Hargis C.W., Juenger M.C.G. (2012). Understanding expansion in calcium sulfoaluminate–belite cements. Cem. Concr. Res..

[13] Madan Mohan Reddy K., Srimurali M., Bhaskar M., Mohan Reddy K. (2014). Characterization of calcium sulfoaluminate cement. Int. J. ChemTech Res..

[14] Aranda M.A.G., De la Torre A.G. (2013). Sulfoaluminate Cement, Eco-Efficient Concrete.

[15] Winnefeld F., Kaufmann J. Concrete produced with calcium sulfoaluminate cement—A potential system for energy and heat storage. Proceedings of the First Middle East Conference on Smart Monitoring, Assessment and Rehabilitation of Civil Structures (SMAR 2011).

[16] Ambroise J., Pera J. (2008). Immobilization of calcium sulfate contained in demolition waste. J. Hazard. Mater..

[17] Hargis C.W., Telesca A., Monteiro P.J.M. (2014). Calcium sulfoaluminate (Ye’elimite) hydration in the presence of gypsum, calcite and vaterite. Cem. Concr. Res..

[18] Martin L.H., Winnefeld F., Müller C.J., Lothenbach B. (2015). Contribution of limestone to the hydration of calcium sulfoaluminate cement. Cem. Concr. Compos..

[19] Winnefeld F., Lothenbach B. (2010). Hydration of calcium sulfoaluminate cements—Experimental findings and thermodynamic modelling. Cem. Concr. Res..

[20] Berger S., Coumes C.C.D., Le Bescop P., Damidot D. (2011). Influence of a thermal cycle at early age on the hydration of calcium sulphoaluminate cements with variable gypsum contents. Cem. Concr. Res..

[21] Li P., Gao X., Wang K., Tam V.W.Y., Li W. (2020). Hydration mechanism and early frost resistance of calcium sulfoaluminate cement concrete. Constr. Build. Mater..

[22] Batog M., Synowiec K. (2017). Cement I Spoiwa Specjalne Zawierające Klinkier Siarczanoglinianowy (Cement and Special Binders Containing Sulphoaluminate Clinker).

[23] Qin L., Gao X., Zhang A. (2018). Potential application of Portland cement-calcium sulfoaluminate cement blends to avoid early age frost damage. Constr. Build. Mater..

[24] Ramanathan S., Halee B., Suraneni P. (2020). Effect of calcium sulfoaluminate cement prehydration on hydration and strength gain of calcium sulfoaluminate cement-ordinary portland cement mixtures. Cem. Concr. Compos..

[25] Li W. (2018). The properties and hydration of portland cement containing calcium sulfoaluminate cement. Ceram. Silik..

[26] Dovál M., Palou M., Kovár V. (2005). Heat evolution and mechanism of hydration in CaO-Al_2_O_3_-SO_3_ system. Ceram. Silik..

[27] Jeong Y., Hargis C.W., Kang H., Chun S.C., Moon J. (2019). The effect of elevated curing temperatures on high Ye’elimite calcium sulfoaluminate cement mortars. Materials.

[28] Wang P., Li N., Xu L. (2017). Hydration evolution and compressive strength of calcium sulphoaluminate cement constantly cured over the temperature range of 0 to 80 °C. Cem. Concr. Res..

[29] (2011). BS EN 197-1:2011 cement. Composition, Specifications and Conformity Criteria for Common Cements.

[30] Klemczak B., Batog M., Giergiczny Z., Żmij A. (2018). Complex effect of concrete composition on the thermo-mechanical behaviour of mass concrete. Materials.

[31] Klemczak B., Żmij A. (2019). Reliability of standard methods for evaluating the early-age cracking risk of thermal-shrinkage origin in concrete walls. Constr. Build. Mater..

[32] Hansen P.F., Pedersen E.J. (1977). Maturity computer for controlling curing and hardening of concrete. Nord. Betongfoerbundet.

[33] D’Aloia L., Chanvillard G. (2002). Determining the “apparent” activation energy of concrete: *E*_a_—Numerical simulations of the heat of hydration of cement. Cem. Concr. Res..

[34] Schindler A.K., Folliard K.J. (2005). Heat of hydration models for cementitious materials. ACI Mater. J..

[35] Poole J.L., Riding K.A., Folliard K.J., Juenger M.C.G., Schindler A.K. (2007). Methods for calculating activation energy for Portland cement. ACI Mater. J..

[36] Wilińska I., Pacewska B. (2018). Influence of selected activating methods on hydration processes of mixtures containing high and very high amount of fly ash. J. Therm. Anal. Calorim..

[37] (2018). CEN, EN 196-11:2018 Methods of testing cement. Heat of Hydration. Isothermal Conduction Calorimetry Method.

[38] Glasstone S., Laidler K., Eyring H. (1941). The Theory of Rate Processes.

[39] Zákoutský J., Tydlitát V., Černý R. (2012). Effect of temperature on the early-stage hydration characteristics of Portland cement: A large-volume calorimetric study. Constr. Build. Mater..

[40] Klemczak B., Batog M. (2016). Heat of hydration of low-clinker cements. Pt. 1: Semi-adiabatic and isothermal tests at different temperature. J. Therm. Anal. Calorim..

[41] Han F., Zhang Z., Wang D., Yan P. (2015). Hydration kinetics of composite binder containing slag at different temperatures. J. Therm. Anal. Calorim..

[42] Wirquin E., Broda M., Duthoit B. (2002). Determination of the apparent activation energy of one concrete by calorimetric and me-chanical means Influence of a superplasticizer. Cem. Concr. Res..

[43] Kada-Benameur H., Wirquin E., Duthoit B. (2000). Determination of apparent activation energy of concrete by isothermal calorimetry. Cem. Concr. Res..

[44] Broda M., Wirquin E., Duthoit B. (2002). Conception of an isothermal calorimeter for concrete—Determination of the apparent activation energy. Mater. Struct..

[45] Klemczak B., Batog M. (2016). Heat of hydration of low-clinker cements. Pt. 2: Determination of apparent activation energy and validity of the equivalent age approach. J. Therm. Anal. Calorim..

[46] Zhang J., Ke G., Liu Y. (2021). Early hydration heat of calcium sulfoaluminate cement with influences of supplementary cementitious materials and water to binder ratio. Materials.

[47] Winnefeld F., Barlag S. (2010). Calorimetric and thermogravimetric study on the influence of calcium sulfate on the hydration of ye’elimite. J. Therm. Anal. Calorim..

[48] Bobrowski A., Nocuń-Wczelik W., Gawlicki M., Łagosz A.K., Łój G. (2015). Cement: Metody Badań, Wybrane Kierunki Stosowania. (Cement: Testing Methods, Chosen Uses).

[49] Neville A.M. (2012). Properties of Concrete.

